# Data on the mode of binding between avenanthramides and IKKβ domains in a docking model

**DOI:** 10.1016/j.dib.2018.02.001

**Published:** 2018-02-06

**Authors:** Chounghun Kang, Woo Shik Shin, Dongwook Yeo, Wonchung Lim, Li Li Ji

**Affiliations:** a Laboratory of Physiological Hygiene and Exercise Science, School of Kinesiology, University of Minnesota, MN 55455, United States; b Department of Physical Education, Inha University, South Korea; c David Geffen School of Medicine, University of California, Los Angeles, United States; d Department of Sports Medicine, College of Health Science, Cheongju University, South Korea

## Abstract

The data presented in this article are related to the research paper entitled “Anti-inflammatory effect of avenanthramides via NF-κB pathways in C2C12 skeletal muscle cells.” (Kang et al., in press) [1] This article includes experimental procedures used to analyze the mode of binding between and IkB kinase (IKKβ) and avenanthramides which are a group of phenolic alkaloids found in oats. The protein-ligand docking and the computer simulation method of molecular dynamics (MD) for studying the physical interactions of molecules were performed.

**Specifications Table**

**Table:** 

Subject area	*Cell Metabolism*
More specific subject area	*Phytochemicals and Inflammatory responses*
Type of data	*Image, Figure*
How data was acquired	Molecular Docking (Schrodinger modeling suite package; Maestro 9.3G, Prime 3.1, Macromodel 9.9, Desmond 3.1; Schrodinger, LLC: NY, USA 2012)
	Molecular Dynamics (MD) Simulation (DESMOND ver. 3.1)
Data format	*Analyzed data*
Experimental factors	*The crystallographic structures of the targeted IKKβ (PDB code:* 3RZF*) with bound inhibitors were used as the starting point for examining the potential mode of binding of avenanthramides.*
Experimental features	*Very brief experimental description*
Data source location	*Department of Physical Education, Inha University, Incheon 22212 Republic of Korea*
Data accessibility	*Data is with this article.*

**Value of the data**

• The molecule docking model first describes the mode of binding between Avns and IKKβ molecular domains.
• Molecular dynamic (MD) simulations were carried out to establish the protein-ligand complex.
• In summary, protein-ligand docking and molecular dynamics simulations methods were performed to understand the potential structure and the nature of molecular clusters with fine interactions with IKKβ.

## 1 Data

**Fig. 1:**
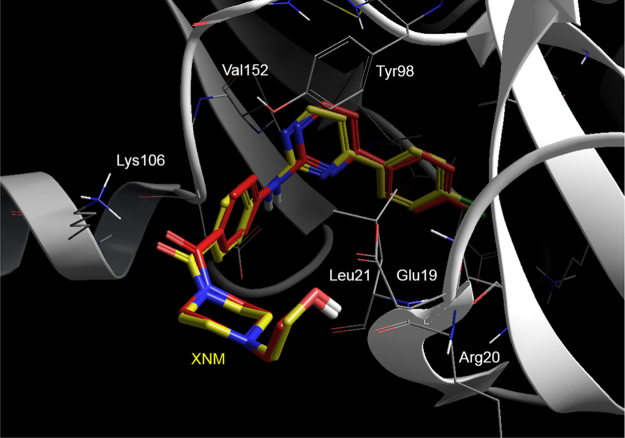
Successful re-docking (red) of XNM back into its original X-ray structures (yellow) by the Standard Precision protocol of Schrodinger's Glide v5.6 used in the docking study.

**Fig. 2:**
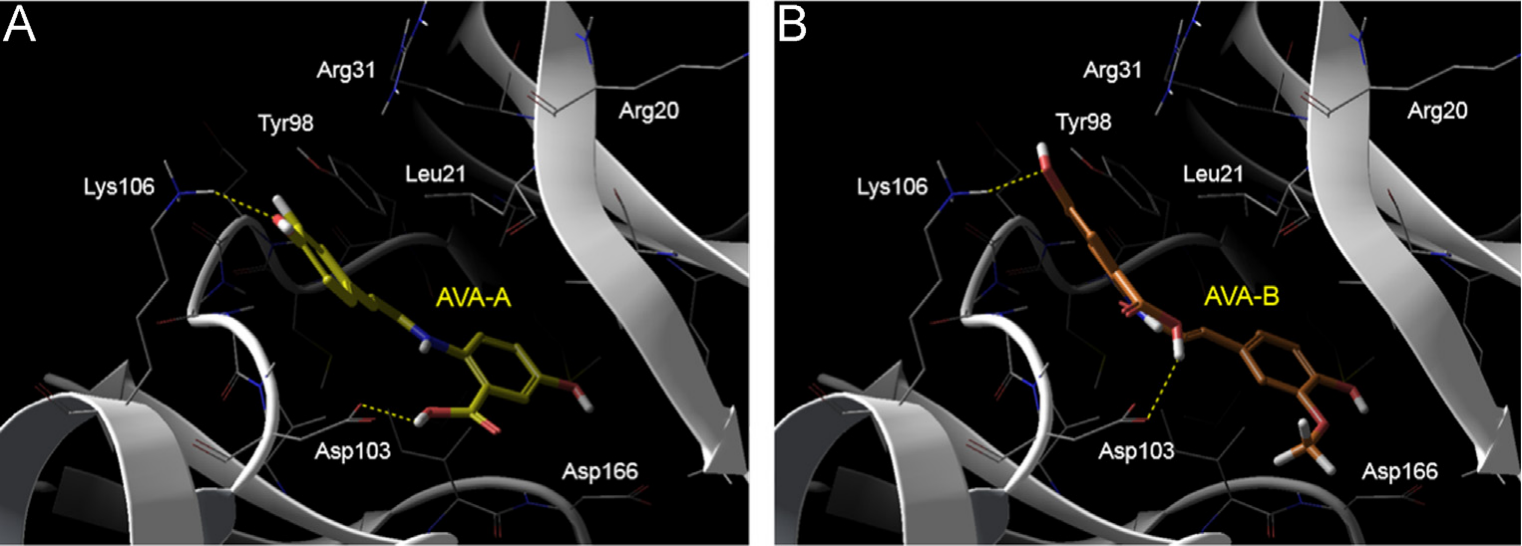
Observed docking poses of (A) AvnA with docking score − 4.81 and (B) AvnB with docking score − 5.13 within the ligand binding IKKβ KD domain.

**Fig. 3:**
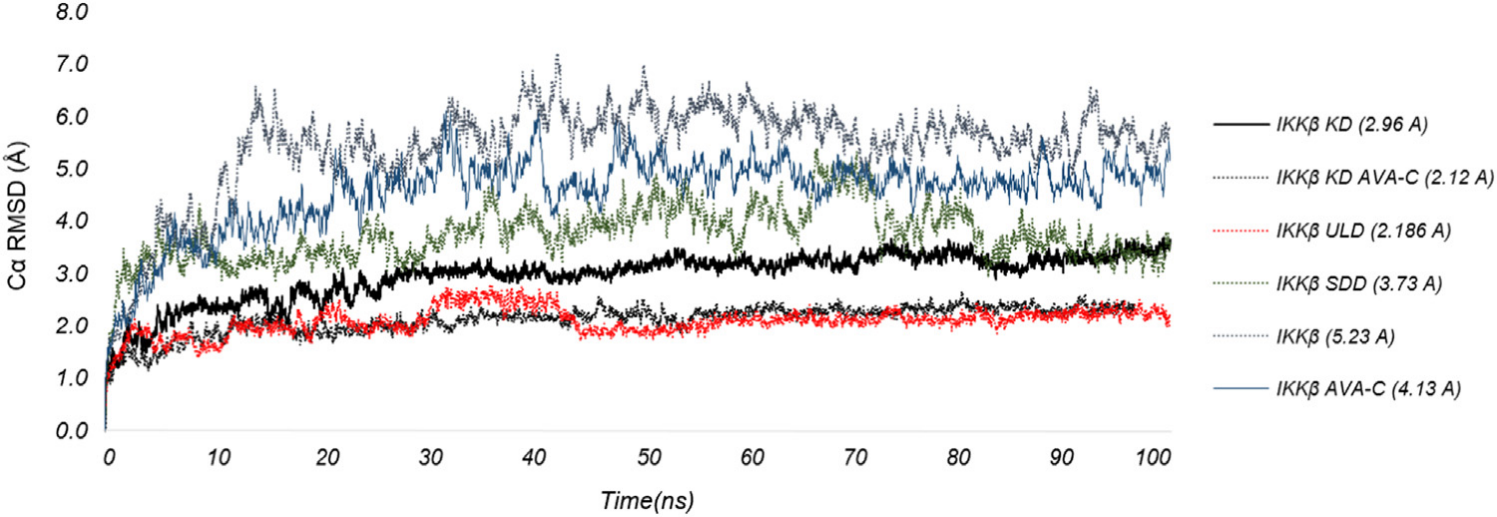
The plot of C_α_RMSD for the KD (black), ULD (red) and SDD (green) domains of IKKβ in the complex to XNM and AvnC over the course of 100 ns MD simulation. Kinase domain, KD; a ubiquitin-like domain, ULD; and an elongated, a-helical scaffold/dimerization domain, SDD.

The data presented in this article are supportive to the data presented in [1]. Avns has emerged as a widely used natural compound in foods that can control cellular defense against oxidation and inflammation due to inhibition of NF-κB [2], [3]. However, despite much interest and considerable research on Avns, the molecular mechanism by which Avns inhibits the NF-κB pathway is not yet clear. The data in this article demonstrate protein-ligand docking (Fig. 1, Fig. 2) and MD stimulation experiments (Fig. 3) to support that the anti-inflammatory function of Avns is conferred by allosteric inhibition of IKKβ, a major activator of the NF-κB pathway [4].

## 2 Experimental design, materials and methods

### 2.1 Molecular docking

A standard protocol for protein-ligand docking studies was used. In short, all docking studies were performed using the Schrodinger modeling suite package (Maestro 9.3G, Prime 3.1, Macromodel 9.9, Desmond 3.1; Schrodinger, LLC: NY, USA 2012). The crystallographic structure of the targeted IKKβ (PDB code: 3RZF) with bound inhibitors was used as a starting point to study the potential mode of Avns binding [5]. All crystallographic water and ions were removed prior to addition of the missing hydrogen atoms, depending on the ionizable state at physiological pH. The energy of the protein structure was minimized using the OPLS-AA 2005 force field to optimize all hydrogen bonding interactions [6]. Using Schrodinger's standard Glide v5.6 protocol, Avns inhibitors were constructed and docked to protein structures without restrictions. To identify key residues associated with molecular recognition, the interaction energy per residue between each docked ligand to residues within 12 Å of the target binding site was evaluated with a constant dielectric constant of 4 [1].

### 2.2 Molecular dynamics (MD) simulation

MD simulations were performed for IKKβ in complex with the structurally solved inhibitors. Each system was solvated in a cubic box with positive TIP3P water [7] and nutrient ions composed of a solvent buffer zone at the 10 Å edge of the composite. A 100 ns simulation was performed on the docking model using the OPLS-AA 2005 force field under isoelectric isothermal (NPT) conditions at 300 K using DESMOND ver 3.1 (Research DES, Desmond Molecular Dynamics System, NY, USA 2008). The stability of the simulation was evaluated by monitoring the CαRMSD (Root-mean-square deviation of α-carbon) with respect to the minimized starting structure. For IKKβ consisting of the kinase domain (KD), ubiquitin-like domain (ULD) and scaffold/dimerization domain (SDD), CαRMSD was evaluated for the ligand binding KD domain [1]. This work was supported by INHA UNIVERSITY Research Grant.
