# Supplementary material for: Data on the mode of binding between avenanthramides and IKKβ domains in a docking model
Source: Data Brief. 2018 Feb 6;17:994–7. doi: 10.1016/j.dib.2018.02.001 (PMC5988504; doi:10.1016/j.dib.2018.02.001)
Supplement: Supplementary file 1 — Transparency document [file mmc1.doc]

Declarations of interest
Their is no conflict of interest.
